# Digital Light Processing 3D‐Printed Silica Aerogel and as a Versatile Host Framework for High‐Performance Functional Nanocomposites

**DOI:** 10.1002/advs.202204906

**Published:** 2022-10-26

**Authors:** Weizhi Zou, Zhen Wang, Zhenchao Qian, Jian Xu, Ning Zhao

**Affiliations:** ^1^ Beijing National Laboratory for Molecular Sciences Laboratory of Polymer Physics and Chemistry Institute of Chemistry Chinese Academy of Sciences Zhongguancun North First Street 2 Beijing 100190 P. R. China; ^2^ University of Chinese Academy of Sciences Beijing 100049 P. R. China

**Keywords:** additive manufacturing, hierarchical pore, interpenetrating phase nanocomposite, mechanical performance, sol‐gel transition

## Abstract

Vat‐photopolymerization‐based 3D printing enables on‐demand construction of customized objects with scalable production capacity and high precision. Herein, the sol‐gel process for aerogels with digital light processing 3D printing to produce advanced functional materials possessing hierarchical pore structures and complex shapes is combined. It has revealed the temporal evolution of the photorheological behavior of acrylate‐modified silica sols in an acid‐base catalytic procedure, and confirmed that silica aerogels can be fabricated with very low acrylate content. The resulting aerogels are thermostable with intrinsic silica contents, skeletal densities, and physical characteristics similar to those of commercial silica aerogels yet distinct mechanical behaviors. More importantly, the printed silica aerogels can be used as a versatile nanoengineering platform to produce high‐performance and multifunctional interpenetrating phase nanocomposites with complex shapes through programmable post‐printing processes. Epoxy‐based nanocomposites possessing excellent mechanical performance, ionogel‐based conductive nanocomposites with decoupled electrical and mechanical properties, and anti‐swelling hydrogel‐based nanocomposites are demonstrated. The results of this study offer new guidelines for the design and fabrication of novel materials by additive manufacturing.

## Graphical abstract

An acid‐base catalyzed sol‐gel strategy that enables fabrication of silica aerogels with very low acrylate content by a commercial digital light processing (DLP) printer has been developed. The physical characteristics of the printed aerogels are comparable to those of commercial silica aerogels and possess distinct mechanical behaviors. Combining the printed aerogels as frameworks with various materials, nanocomposites with notable mechanical properties and multifunctionality were demonstrated.

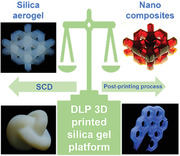

## 1 Introduction

As a revolutionary manufacturing technique, 3D printing including stereolithography/digital light processing (SLA/DLP, vat‐photopolymerization‐based), powder bed fusion, inkjet, and fused deposition modeling/direct ink writing (FDM/DIW, extrusion‐based) and so on, has facilitated innovations in materials science due to its features of high accuracy, low material cost, multi‐material compatibility, and ability to rapid build digitally designed complex objects on demand.^[^ ^1^, ^2^, ^3^, ^4^ ^]^ Combinations of sol‐gel methods and suitable drying processes (e.g., freeze‐drying and supercritical drying) with 3D printing protocols have been developed to engineer desirable carbon,^[^ ^5^, ^6^ ^]^ biomass,^[^ ^7^ ^]^ metal,^[^ ^8^ ^]^ and silica‐based aerogels^[^ ^9^, ^10^, ^11^ ^]^ for emerging applications in lightweight support materials, energy storage, catalysis, sensors, and medicine. Silica aerogel is by far the most widely used type of aerogel because its density can be tuned over a wide range and its characteristics include high porosity, large specific surface area, superior thermal insulation, acoustic impedance, fire resistance, adjustable transparency and high intrinsic modulus suitable for use as a reinforcement for composites.^[^ ^9^, ^12^, ^13^, ^14^, ^15^ ^]^ Zhao et al. employed a shear‐thinning ink, i.e., a slurry of silica aerogel particles in polyethoxydisiloxane precursor sol, to prepare silica aerogels by DIW for the first time.^[^ ^9^ ^]^ Compared to extrusion‐based 3D printing techniques, DLP provides more scalable production capability^[^ ^16^ ^]^ and higher precision (currently reaching the ≈100 nm scale).^[^ ^17^ ^]^ However, DLP relies on the solidification of liquid resins by photopolymerization to construct objects. The introduction of photosensitive groups in the silica precursor to make it photopolymerizable would reduce the intrinsic mechanical properties, specific surface area, and fire resistance of silica‐based materials.^[^ ^18^, ^19^, ^20^ ^]^ Moreover, the removal of the organic components from DLP‐printed silica‐based aerogels inevitably damages the aerogel structure.^[^ ^11^ ^]^

In this study, we developed a two‐step acid‐base catalytic procedure for the fabrication of acrylate‐modified silica sols and reveal the temporal evolution of their photorheological behavior for the first time. When this modified silica sol with a very low acrylate content was close to its gelation point, maintaining a low viscosity enabled a fast and stable photo‐controllable sol‐gel transition that can be used for 3D printing of silica wet gels by a commercial DLP printer. The resultant aerogels showed mesoporous characteristics of high porosity, pore volume and specific surface area, and thermostability, inorganic content, skeletal density, and fire resistance comparable to those of commercial silica aerogels.^[^ ^9^, ^21^ ^]^ Moreover, the DLP‐printed silica aerogel (bulk density of ≈0.15 g cm^−3^) possessed a notable compressive resilience. We further employed the aerogel as a nanomaterial engineering platform to prepare novel nanocomposites with complex shapes through programmable post‐printing processes. The synergistic effect of the silica skeleton and polymer matrices on the mechanical properties as well as the specific functions of the digitally designed nanocomposites were demonstrated.

## 2 Results and Discussion

**Figure 1:**
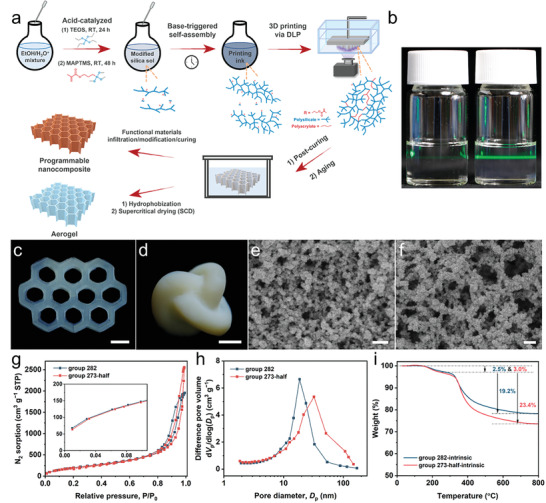
Preparation, morphology and selected physical characteristics of DLP‐printed aerogels. a) Overview of the preparation of DLP‐printed silica aerogels and aerogel‐based programmable multiphase nanocomposites. b) Tyndall effect of the printing ink (group 282) at different preparation stages. Left: the ink in the acid condition (the first stage); Right: the ink in a stable printing stage ready for DLP (after the addition of 0.2 wt% HMTA and heating at 50 °C for 2 h, the second stage). c) DLP‐printed honeycomb‐shaped silica aerogel from group 282 after supercritical drying (SCD) and d) DLP‐printed interlock‐shaped silica aerogel from group 273‐half after SCD. SEM photograph of the obtained aerogel from group 282 e) and from group 273‐half f). g) Sorption/desorption isotherms of N_2_ at 77 K; The inset shows the presence of N_2_ adsorption at a relative pressure (*P*/*P*_0_) close to 0. h) Pore size distributions of the aerogels based on the Barrett–Joyner–Halenda (BJH) method. i) Thermogravimetric analysis of the hydrophilic aerogels (intrinsic state without hydrophobization) in the air; The weight loss of the hydrophilic aerogels at <250 °C was attributed to the adsorbed water and a small amount of dye (see Experimental sections) that could not be extracted by SCD. Scale bar: 5 mm in (c) and (d); 100 nm in (e) and (f).

The procedure of DLP printing of silica aerogels and corresponding aerogel‐based nanocomposites is schematically shown in **Figure** **1**a. Tetraethoxysilane (TEOS) was reacted under acidic conditions at room temperature to form polysilicic acid. Then, the silane coupling agent 3‐methacryloxypropyl trimethoxysilane (MAPTMS) was added to graft acrylate onto the polysilicic acid. The corresponding naming conventions of the obtained acrylate‐modified silica sols are shown in Table S1, Supporting Information. For example, group 282 indicates that the volume ratio of TEOS and MAPTEMS in the sol was 28:2. In the modified silica sol, an organic base of hexamethylenetetramine (HMTA) was added to promote further condensation and initiate the self‐gelation process. Compared with adding the two precursors simultaneously or adding MAPTMS followed by TEOS, the steric hindrance effect of the methacrylate groups in the above reaction sequence extended the self‐gelation time, which provided a long pot life for 3D printing but did not greatly hamper the formation of the Si‐O‐Si network through condensation (Figure S1, Supporting Information). The as‐formed silica sol can be used as a printing ink for DLP after incubation for a specific period, and the printed wet gels either were supercritically dried to obtain the corresponding aerogels or used directly to form interpenetrating phase nanocomposites with good shape fidelity through programmable post‐printing processes.

The photocuring behaviors of the modified silica sols before self‐gelation (i.e., the sol loses flowability after the self‐gelation time *t*_sg_) were studied by photorheology. As shown in Figure S2, Supporting Information, after the addition of 1 wt% HMTA in groups 237–273, the photo‐induced gelation time (*t*_g_, after this point, the storage modulus *G′* under irradiation starts to be significantly larger than the loss modulus *G′′*) decreased, and the photo‐crosslinked plateau storage modulus (*G′*_plateau_) increased rapidly at first and then changed slowly with incubation time. To minimize the occurrence of significant gradient differences in the structure and composition of the printed objects, the sols with relatively unchanged *t*_g_ and *G′*_plateau_ were used for DLP and a stable printing window (highlighted as blue areas in Figure S2 and S3) could be obtained. The small amount of acrylate in group 273 did not provide a sufficient steric hindrance effect, leading to rapid self‐gelation under 1 wt% HMTA condition (*t*_sg_ approximately 2 h, Figure S2c) and thus, a short printing window for DLP. Reducing the concentration of HMTA from 1 to 0.2 wt% retarded the self‐gelation process, and the sol could undergo a transition from non‐photocurable to photocurable with incubation time, even at concentrations as low as 1.5 wt% MAPTMS (group 291, the lowest concentration in our study, Figure S3). However, this system did not exhibit a stable printing stage before self‐gelation (*G′*_plateau_ increased observably with time). When the concentration of MAPTMS was slightly increased to 2.9 (group 282) and 4.4 wt% (group 273), the stable printing stages increased to ≈4 and 9 h, respectively. To obtain sufficiently printable time and strong wet gel during DLP, we chose group 282 and diluted group 273 (half of the original concentration, named 273‐half) for the subsequent preparation of aerogels with different bulk densities (see Experimental sections; **Table** **1**) and shortened the incubation time of the sols by heating (Figure S4, Supporting Information).

**Table 1:** Physical characteristics of DLP‐printed silica aerogels

Property sample	*ρ*_bulk_ [g cm^−3^]	*ρ*_skeletal_ [g cm^−3^]	Porosity^a)^ [%]	*V*_total_ ^b)^ [cm^3^ g^−1^]	*S*_BET_ [m^2^ g^−1^]	*V*_p,BJH_ [cm^3^ g^−1^]	*D*_p,BJH_ [nm]	*λ* ^c)^ [mW m^−1^ K^−1^]
273‐half	0.145	1.86	92.2	6.36	771	3.97	15.5	26.7
282	0.266	1.89	85.9	3.23	783	3.01	12.6	35.2

^a)^ Porosity was calculated by 1 − *ρ*_*bulk*_/*ρ*_*skeletal*_;

^b)^ *V*_total_ was calculated by 1/*ρ*_*bulk*_ − 1/*ρ*_*skeletal*_;

^c)^ *λ* is the thermal conductivity.

The shift from base‐initiated self‐gelation to a controlled photocuring process could be interpreted by bond percolation theory, which suggested that the formation of spanning aggregates (i.e., infinitely large molecular clusters) induced the sol‐gel transition of a system.^[^ ^22^, ^23^ ^]^ As the pH increased, the silanol groups in the middle of the polymer chains were deprotonated, since they were more acidic than those at the end of the chains, which induced further condensation through a nucleophilic mechanism and thus generated larger and more highly branched aggregates (Figure 1a).^[^ ^23^, ^24^ ^]^ Intuitively, as demonstrated in Figure 1b, when reaching the stable printing stage for DLP, the printing ink exhibited a more pronounced Tyndall effect than that under acidic conditions since the grown silicate aggregates caused stronger light scattering. During the formation of spanning aggregates through spontaneous Si‐O‐Si bridge bonding, the acrylate group not only provided a steric hindrance effect but also allowed for the premature spanning of aggregates to induce gelation by photopolymerization. When the acrylate content decreased, more Si‐O‐Si bonds were required to achieve gelation under photopolymerization (i.e., more incubation time), and thus, the printable time was reduced. In addition, when the spanning aggregates had not yet formed by Si‐O‐Si bridge bonding, the percolation theory suggested for no significant increase in the apparent viscosity of the sol until near its self‐gelation point, as evidenced from the viscosity measurements (Figure S5, Supporting Information). This meant that the sol could maintain good flowability during the stable printing stage, which was crucial for DLP.^[^ ^2^ ^]^ It should be noted that in this strategy the pot life of the printing ink is limited by its self‐gelation time *t*_sg_, thus means the printing ink loses flowability and cannot be printed after *t*_sg_. Our strategy of employing a spontaneous sol‐gel process to weaken the overreliance on solidification by the photopolymerization of acrylates in DLP can be extended to a wide range of metal alkoxide precursors. In principle, well‐established nonphotoinduced sol‐gel processes can be matched to vat photopolymerization methods of 3D printing with the assistance of small amounts of photopolymerizable groups, greatly expanding the range of raw materials available for additive manufacturing.

We printed a honeycomb‐shaped (group 282) object and an interlock‐ring‐shaped (group 273‐half) object using a commercial DLP printer (Figure 1c,d). The printed layers in the object merged without voids (Figure S6, Supporting Information). The hydrophobic treatment of the skeleton enabled superhydrophobicity of the objects after SCD (Figures S7 and S8, Supporting Information). The aerogel from group 282 had a bluish and transparent appearance, a typical optical characteristic of pure silica aerogel due to Rayleigh scattering (Figure 1c). SEM image confirmed the presence of a homogeneous, pearl‐necklace‐like network, in which the average size of the skeleton was approximately 25 ± 6 nm (Figure 1e and Figure S9a, Supporting Information). In contrast, the aerogel from group 273‐half was opaque and showed a sponge‐like network, and its skeleton had a higher aspect ratio and larger size (approximately 42 ± 10 nm, Figure 1f), which reduced the transmittance by enhancing the extinction coefficient (Figure S9b,c, Supporting Information). The relatively low elastic modulus of the wet gel and higher organic content in group 273‐half might allow the polymers to aggregate and grow into a more robust skeleton, while group 282 underwent stronger kinetic freezing during phase separation due to a higher degree of crosslinking and thus had a smaller skeleton.^[^ ^23^ ^]^ The resulting aerogels demonstrated a combined type II/IV isotherm with a distinct hysteresis loop (Figure 1g), which was a characteristic of the capillary condensation occurring in the mesopores. Pore size distribution based on the BJH method indicates the presence of meso‐ and macro‐scale pores, and the corresponding average pore sizes were 12.6 and 15.5 nm for samples from group 282 and 273‐half, respectively (Figure 1h and Table 1). The inset of Figure 1g reveals that some N_2_ gas molecules adsorbed at *P∕P_0_* →0, implying that micropores (<2 nm) were also present in the aerogels.^[^ ^25^ ^]^ According to calculations by the BET method, the values of the specific surface area (*S*_BET_) were up to 783 m^2^ g^−1^ (group 282) and 771 m^2^ g^−1^ (273‐half), which were larger than the typical value of silica aerogel,^[^ ^13^ ^]^ ≈600 m^2^ g^−1^.

Benefiting from the very low content of acrylate, thermogravimetric analysis (TGA, Figure 1i) showed that the thermal decomposition temperature (*T*_onset_) was approximately 300 °C and the intrinsic inorganic silica contents (*W*_silica_) of these aerogels were as high as 80.8 and 76.6 wt%, respectively. The skeletal densities (*ρ*_skeletal_) of group 282 and 273‐half aerogels were determined to be 1.89 and 1.86 g cm^−3^, respectively (Table 1). The above results approached the properties of commercial silica aerogel (*T*_onset_ ≈350 °C, *W*_silica_ ≈90 wt% and *ρ*_skeletal_ ≈2 g cm^−3^).^[^ ^9^, ^21^ ^] 13^C/^29^Si solid‐state nuclear magnetic resonance spectra demonstrated the presence of both polymethacrylate and silica networks in the printed silica aerogels (Figure S10, Supporting Information). High‐resolution transmission electron microscopy confirmed the absence of microphase separation (Figure S11, Supporting Information).^[^ ^26^ ^]^ Moreover, polymethacrylate did not affect the formation of the silica moiety since the Si‐O‐Si bond angle (*θ*) was 148.3° and 146.9° in group 282 and 273‐half aerogels, respectively (Figure S10, Supporting Information), representing a typical relaxed silica network without heat treatment.^[^ ^18^ ^]^

**Figure 2:**
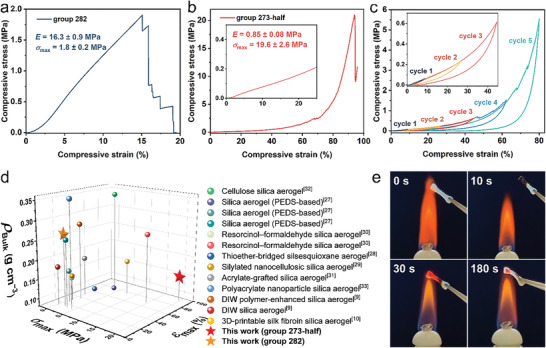
Mechanical properties and fire‐retardant properties of DLP‐printed silica aerogels. a) Uniaxial compressive stress–strain curve of the DLP‐printed silica aerogel from group 282 and the compressive modulus (*E*) and the maximum compressive strength (fracture strength, *σ*_max_). b) Uniaxial compressive stress–strain curve of the DLP‐printed silica aerogel from group 273‐half and the obtained *E* and *σ*_max_. c) Cycling compressive stress–strain curves with progressively larger strain amplitudes of the DLP‐printed silica aerogel from group 273‐half. The inset shows the first 3 loading–unloading cycles. d) Comparison of DLP‐printed silica aerogels and various reported silica‐based aerogels: bulk density and mechanical properties (including typical values of *σ*_max_ and the corresponding fracture strain *ε*_max_). e) Photographs of an ablation test of a printed dumbbell‐shaped aerogel from group 282.

The group 282 aerogel with a pearl‐necklace‐like skeleton exhibited brittle fracture behavior under uniaxial compression, which was similar to that of pure silica aerogels at a bulk density of ≈0.25 g cm^−3^ (**Figure** **2**a,d).^[^ ^27^ ^]^ The sample of group 273‐half presented an unusual mechanical performance compared to the reported pure/hybrid silica aerogels (Figure 2d).^[^ ^9^, ^10^, ^27^, ^28^, ^29^, ^30^, ^31^, ^32^, ^33^ ^]^ It had a significant resilience even at ≈80% compressive strain, could be compressed by more than 90% without noticeable fractures, and exhibited significant failure only after compressive strain at ≈95% (Figure 2b,c). This resilience was also reflected in high tolerance to fracture under cyclic compression‐decompression testing at ≈45% strain for 60 cycles (Figure S12, Supporting Information). Compared to the pearl‐necklace‐like structure that was prone to produce stress concentrations among the secondary particles on the skeleton and brittle fracture, the robust sponge‐like ligaments better distributed stresses to withstand buckling deformation (e.g., inward folding to the pores) during compression (Figure 2c). Notably, although pure silica aerogels became compressible (compressive strain >80%) when their densities were below ≈0.1 g cm^−3^, they underwent plastic deformation and could not spring back after decompression.^[^ ^27^ ^]^ It implies that the introduction of a small amount of polyacrylate in the molecular network softened the rigid silica network. Indeed, introducing polysilsesquioxanes into the siloxane network to reduce the Si‐O‐Si crosslinking density is an effective strategy for fabricating silica‐based aerogels with impressive compression flexibility.^[^ ^34^, ^35^ ^]^ However, the skeletal density of these aerogels was only ≈1.1–1.4 g cm^−3^, indicating that their network was close to a ductile organic polymer network. In our case, the skeletal density was ≈1.9 g cm^−3^, and the network was more rigid and closer to an inorganic silica network.

High mesopore volume and tortuous three‐dimensional nanostructure weakened gas phase and solid phase heat transfer in the aerogels,^[^ ^9^, ^34^ ^]^ and low thermal conductivities were obtained (Table 1). Therefore, the DLP‐printed aerogel objects can be used as effective thermal insulators (Figure S13, Supporting Information). Because of their high inorganic content, their fire‐retardant properties were preserved. Printed samples self‐extinguished within 10 s after being ignited, and transparent silica formed after calcination (Figure 2e and Figure S14, Supporting Information).

**Figure 3:**
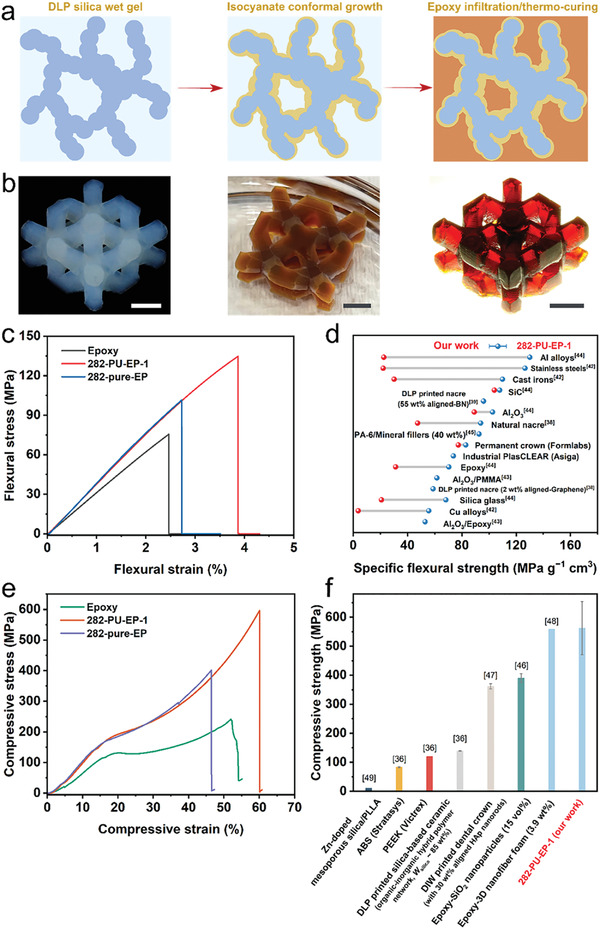
Preparation, morphology, and mechanical properties of epoxy‐based interpenetrating phase nanocomposites. a) Overview of the preparation of isocyanate‐reinforced DLP‐printed silica gel and corresponding nanocomposites based on modified gel skeletons. b) Digital photographs of the printed silica aerogel from group 282 with a diagonal structure (left), the isocyanate‐modified silica wet gels (middle), and the corresponding nanocomposites (right) with good shape fidelity and transparency after epoxy resin infiltration and thermal curing. c) Three‐point bending (3 PB) stress–strain curves of the pure epoxy resin matrix and epoxy‐based nanocomposites with unmodified (282‐pure‐EP) and isocyanate‐modified (282‐PU‐EP‐1) silica gel backbones and d) comparison of the specific flexural strength of a wide range of materials, of which the data of permanent crown resin (Formlabs) and industrial plasCLEAR resin (Asiga) were obtained from https://dental‐media.formlabs.com/datasheets/2002482‐TDS‐ENUS‐0.pdf and https://www.asiga.com/media/main/files/materials/Asiga%20Material%20Handbook%20‐%20Manufacturing%20en_US.pdf, respectively. e) Uniaxial compressive stress–strain curves of the pure epoxy resin matrix and epoxy‐based nanocomposites with unmodified (282‐pure‐EP) and isocyanate‐modified (282‐PU‐EP‐1) silica gel backbones and f) comparison of the compressive strength of typical polymers and polymer‐based composites. Scale bar: 5 mm in (b).

Although 3D printing using vat photopolymerization has been developed for more than 30 years from prototyping to large‐scale manufacturing, the poor mechanical properties of photocurable polymeric materials limit their industrial applications.^[^ ^2^ ^]^ To solve this problem, previous studies have obtained high‐performance materials by 1) designing organic–inorganic hybrid double cross‐linked networks^[^ ^36^ ^]^ or dual‐cured polymer interpenetrating networks at the molecular level^[^ ^37^ ^]^ and 2) preparing bioinspired anisotropic nanocomposites.^[^ ^38^, ^39^ ^]^ In nature, distinguished biological nanocomposites (e.g., tooth, nacre, and bone) adopt submicrometer‐scale three‐dimensional interlocking of soft (polymer)/hard (inorganic framework) phases to achieve outstanding strength and toughness through synergetic load sharing and energy dissipation.^[^ ^40^ ^]^ Inspired by nature, we used the 3D skeleton of a DLP‐printed silica aerogel as a host framework to prepare high‐performance nanocomposites with complex shapes. More importantly, since the preparation process was performed after DLP printing (Figure 1a), the material selection was decoupled from photocurable resins, and epoxy resin with excellent properties that are widely used in conventional composites was adopted. To maximize the mechanical responses of the silica skeleton, we employed isocyanates to strengthen the DLP‐printed wet gels and then filled the gels with epoxy‐anhydride resin via solvent exchange, followed by thermal curing to obtain nanocomposites (**Figure** **3**a; see Experimental Section). Isocyanates reacted with Si‐OH groups on the skeleton surface and subsequently self‐hydrolytically condensed to produce polyurethane/polyurea on the gel skeleton conformally, which acted as “cross‐linked tethers” to fill the mesopores and widen the necks on the skeleton (Figure S15 and Table S2, Supporting Information).^[^ ^41^ ^]^ Thus, the stress concentrations of aerogel skeletons from group 282 could be reduced to prevent premature failure by external forces (Figures S16 and S17, Supporting Information). The strong capillary force of the pores of the gel network and the low solidification shrinkage of epoxy resin ensured that the 3D objects maintained good shape from DLP printing after epoxy infiltration and thermal curing (Figure 3b). Meanwhile, the as‐formed nanocomposites demonstrated good transparency since the polymer phase eliminated the refractive index difference at the original air/skeleton interface in the aerogel (Figure S18, Supporting Information). The as‐prepared nanocomposites were named according to the epoxy content (*f*_e_, assuming equal to the porosity of the modified aerogels, 282‐PU) as 282‐PU‐EP‐1 (*f*_e_ = 62.3%), 282‐PU‐EP‐2 (*f*_e_ = 47.5%), and 282‐PU‐EP‐3 (*f*_e_ = 33.0%) (Table S2, Supporting Information). Compared to the cured epoxy matrix (75 ± 11 MPa), composites with an unmodified backbone (282‐pure‐EP, *f*_e_ = 85.9%) had flexural strength of 97 ± 9 MPa, and 282‐PU‐EP‐1 had flexural strength of 132 ± 8 MPa (Figure 3c). Even though the volume of epoxy in 282‐PU‐EP‐3 was only 33%, its strength reached 126 ± 8 MPa (Figure S19, Supporting Information). Notably, the energy absorption capacity of the materials (the area under the stress–strain curve) increased from 0.98 ± 0.35 MJ cm^−3^ for the epoxy matrix to 2.72 ± 0.39 MJ cm^−3^ for 282‐PU‐EP‐1. The increase in the flexural strength and strain from 282‐pure‐EP to 282‐PU‐EP‐1 benefited from the better toughness of the isocyanate‐modified aerogel network. Considering that the bulk density of the 282‐PU‐EP‐1 composite was only 1.24 g cm^−3^, its specific strength exceeded that of most metallic materials, ceramics, typical polymer‐based composites, and natural nacres, and it exhibited characteristics of being lightweight and having high mechanical strength (Figure 3d).^[^ ^38^, ^39^, ^42^, ^43^, ^44^, ^45^ ^]^ A significant improvement was also observed in compressive performance (Figure 3e and Figure S20, Supporting Information). The compressive strength increased to 562 ± 92 MPa for 282‐PU‐EP‐1 with no sacrifice in compressive strain. These materials were different from epoxy composites reinforced by a relatively rigid skeleton (282‐pure‐EP) or silica nanoparticles, which were shown to increase strength but decrease strain.^[^ ^46^ ^]^ It also indicates that the introduction of polyurethane/polyurea phase further enhanced the toughness of the nanocomposites. As demonstrated in Figure 3f, compared to nanofiller reinforcement strategies or designing organic–inorganic hybrid double cross‐linked polymer networks, nanocomposites with bicontinuous interpenetrating phases achieved significant mechanical superiority through synergistic load sharing and energy dissipation.^[^ ^36^, ^46^, ^47^, ^48^, ^49^ ^]^ Of particular note is that, when striving for high mechanical properties, the dispersion problems and high viscosity associated with high nanofiller content in resins limited processability using vat photopolymerization methods of 3D printing.^[^ ^2^, ^50^ ^]^ In contrast, we offer a post‐printing cast‐in‐place strategy that is compatible with commercial DLP printers and provides a powerful and cost‐efficient solution for the additive manufacturing of high‐performance nanocomposites.

**Figure 4:**
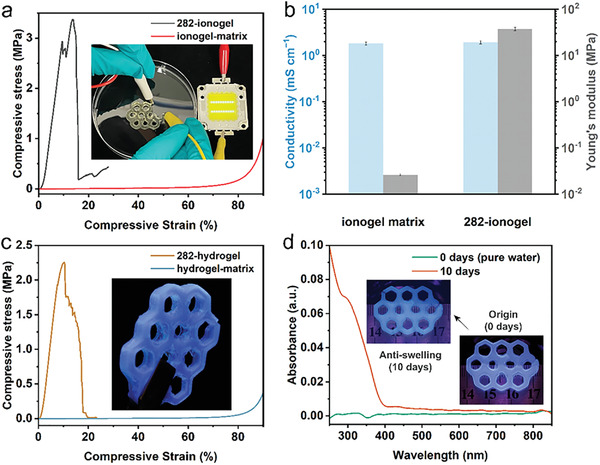
Multifunctionality of ionogel and hydrogel‐based interpenetrating phase nanocomposites. a) Compressive properties of the 282‐ionogel nanocomposite and ionogel matrix. The inset shows the 282‐ionogel nanocomposite in a damaged state still able to light the LED bulb. b) Comparison of Young's modulus and ionic conductivity of the nanocomposite and ionogel matrix. c) Compressive properties of the 282‐hydrogel nanocomposite and hydrogel matrix. The inset shows a honeycomb‐shaped 282‐hydrogel nanocomposite loaded with fluorescent dye emitting bluish fluorescence under UV light (385 nm). d) The absorbance of the aqueous solution demonstrated the release of fluorescent molecules from the nanocomposite into water. The inset shows no significant change in the size of the nanocomposite after 10 days of soaking, indicating remarkable anti‐swelling performance.

We also demonstrated that the DLP‐printed silica aerogel can serve as a versatile framework for functional soft materials. The wet gel was filled with conductive ionic liquid ([Emim][NTF_2_])‐acrylate precursor by solvent replacement, and then the gel was photocured to obtain nanocomposites. Since the bicontinuous interpenetrating structure provided both mechanical support and independent ion transport channels, the modulus of the composite increased nearly 1440‐fold compared to the ionogel matrix, while the conductivity remained almost constant (1.81 vs 1.92 mS cm^−1^, **Figure** **4**a,b and Figure S21, Supporting Information). This decoupling of modulus and conductivity successfully circumvented the general requirement for the use of polymer‐based ionogels with weak mechanical properties to realize effective ion transport.^[^ ^15^ ^]^ For biomedical applications, the aerogels could be combined with acrylate‐based hydrogels (water content 80 wt%) loaded with functional components. In this study, we used the fluorophore disodium 4,4′‐bis(2‐sulfonatostyryl)biphenyl as an example. The fluorescer was uniformly loaded in the hydrogel‐silica aerogel nanocomposite, which exhibited swelling resistance and almost no loss of mechanical properties after 10 days of soaking due to the mechanical support provided by the aerogel (Figure 4c,d and Figures S22 and S23, Supporting Information). This performance is crucial for practical applications of hydrogel materials such as load‐bearing implants in the human body.^[^ ^51^ ^]^

## 3 Conclusion

In summary, we introduced a two‐step acid‐base catalyzed sol‐gel transition into DLP printing. This strategy enabled solid‐free fabrication of silica wet gels with very low acrylate content by a commercial DLP printer. After drying, the thermostability, inorganic content, and backbone density of the printed aerogels were comparable to those of commercial silica aerogels. The DLP‐printed silica aerogels not only possessed mesoporous features, high porosity, large pore volume, high specific surface area, and fire resistance but also had excellent compressive resilience at low bulk density. By using the printed silica aerogels as host frameworks for conventional resins and soft materials, nanocomposites with complex shapes and notable mechanical properties were prepared. Our strategy may enable vat‐photopolymerization‐based 3D printing to be available for a broader range of nonphotosensitive materials, greatly expanding the prospects for the application of additive manufacturing for industrial and personal customization.

## 4 Experimental Section

Full experimental details are provided in the Supporting Information.

## Conflict of Interest

The authors declare no conflict of interest.

## Supporting information

Supporting Information

## Data Availability

The data that support the findings of this study are available from the corresponding author upon reasonable request.
