# Supplementary material for: Digital Light Processing 3D‐Printed Silica Aerogel and as a Versatile Host Framework for High‐Performance Functional Nanocomposites
Source: Adv Sci (Weinh). 2022 Oct 26;9(36):2204906. doi: 10.1002/advs.202204906 (PMC9798997; doi:10.1002/advs.202204906)
Supplement: Supplementary file 1 — Supporting Information [file ADVS-9-2204906-s001.pdf]

ADVANCED SCIENCE
Open Access

Supporting Information

for *Adv. Sci.*, DOI 10.1002/advs.202204906

Digital Light Processing 3D-Printed Silica Aerogel and as a Versatile Host Framework for High-Performance Functional Nanocomposites

*Weizhi Zou, Zhen Wang, Zhenchao Qian, Jian Xu and Ning Zhao**

Supporting Information

Digital Light Processing 3D-printed Silica Aerogel and as a Versatile Host Framework for High-performance Functional Nanocomposites

*Weizhi Zou, Zhen Wang, Zhenchao Qian, Jian Xu and Ning Zhao**

Experimental Section

Preparation of acrylate-modified silica sols: Ethanol of 92.5 g and 0.1 mol L^{-1} HCl aqueous solution (pH~1) of 36.2 g were mixed in a round-bottom flask and magnetically stirred for 5 min. TEOS (Acros) was added dropwise to the above solution while stirring, and the reaction was conducted at room temperature for 24 h. Subsequently, MAPTMS (Sigma–Aldrich) was added, and the reaction continued for another 48 h for the formation of acrylate-modified silica sol. The amounts of TEOS and MAPTMS are summarized in Table S1. For example, the volumes of TEOS and MAPTMS were 84 and 6 mL, respectively, for the sol of group 282.

DLP-printed silica aerogels: A typical formulation of printing ink was as follows. In group 282, 0.2 wt% HMTA (Aladdin; dissolved in 1 g equimolar ethanol/water mixture) and 0.335 wt% diphenyl(2,4,6-trimethylbenzoyl)phosphine oxide (TPO, photoinitiator, J&K Scientific) were added, followed by stirring and heating at 50 °C for 2 h. Printing inks were obtained after the sol was cooled to room temperature, and 0.015 wt% dye of phenol red sodium (Innochem) was added. The same procedure was used to prepare the ink of group 273, except 0.5 wt% photoinitiator, and heating time of 3 h were used. Then, the mixture was diluted to half of its original concentration by an ethanol/water mixture (molar ratio 3.8:1) to obtain group 273-half (with 0.0125 wt% dye added). 3D printing was performed with a commercial DLP printer (Asiga Max X27, equipped with a DMD with a pixel resolution of 1920 × 1080 and a 385 nm UV LED lamp with a settled output light intensity of 27 mW cm^{-2}) through a bottom-up layer-by-layer building process in the vertical direction. The printing layer thickness was 100 μm for burn-in layers and 200 μm for normal layers, and the exposure time was approximately 30-40 s. The dye system was changed to a mixture of Rhodamine B (Acros, typical value of 0.03 wt%) and propyl gallate (J&K Scientific, typical value of 0.15 wt%),[1] and the exposure time of the normal layer was adjusted to 13 s with an exposure light intensity of 18 mW cm^{-2} during the printing process of complex hollow gel objects using group 282. After printing, the objects were post-cured at least 30 min in an equimolar ethanol/water mixture with 0.2 wt% HMTA and under irradiation of a 385

nm UV LED lamp with an output power of ~10 mW cm^{-2}, followed by aging in an oven at 80 °C for 5 h. The aged objects were subjected to solvent exchange with ethanol 6 times (8-12 h each) at room temperature and hydrophobic treatment in a hexamethyldisilazane (Acros) solution (15 wt% in ethanol) at 60 °C for 24 h. All samples were dried by a supercritical CO_2 dryer (ShiAnJia (Beijing) Biotechnology). Hydrophilic samples were dried directly after solvent exchange without the hydrophobization treatment.

Isocyanate-modified DLP-printed silica aerogels: The modification followed a previously reported method.[2] In brief, after washing with ethanol (4 times, 8-12 h each) and acetone (4 times, 8-12 h each), the printed wet gels were immersed in a poly(hexamethylene diisocyanate) (Sigma–Aldrich)/acetone solution, equilibrated under frequent agitation for 24 h, heated to 55 °C and held for 48 h. Isocyanate/acetone solutions of 1) 5/95 with a total mass of 20 g, 2) 50/50 with a total mass of 20 g, and 3) 50/50 with a total mass of 50 g were used to control the amount of isocyanate reacted on the skeleton. Different volumes of 3D printed wet gel objects were modified in the isocyanate/acetone solution maintaining the same ratios (*v*/*w*) of gel volume and solution mass. Afterward, the samples were cooled to room temperature and washed with fresh acetone (4 times, 8-12 h each). All isocyanate-modified samples were dried with a supercritical CO_2 dryer.

Silica aerogel-based nanocomposites: For epoxy-silica aerogel nanocomposites, aged pure wet gels and isocyanate-modified wet gels were washed with acetone and then transferred to a new container with epoxy (*N,N*-diglycidyl-4-glycidyloxyaniline, IC510, Indosol Chemie)-anhydride (methylhexahydrophthalic anhydride, MHHPA, Innochem) resin (w_{IC510}/w_{MHHPA}=1) containing 0.25 wt% tris(dimethylaminomethyl)phenol (TCI). The resin was replaced three more times for 24 h each. Next, the samples were removed from the container, excess resin on the surface was wiped off, and the samples were transferred to an oven for gradient thermal curing (90 °C for 2 h and 150 °C for 7 h). The pristine epoxy-anhydride resin was cured under the same curing conditions. For ionogel-silica aerogel nanocomposites, after ethanol washing, the aged DLP-printed

wet gels from group 282 were transferred to ionogel precursor solution (70 wt% ionic liquid 1-ethyl-3-methylimidazolium bis((trifluoromethyl)sulfonyl)imide ([Emim][NTF_2], Bide Pharmatech), 29.4 wt% ethyl acrylate (EA, Alfa Aesar), 0.6 wt% triethylene glycol dimethacrylate (TEGDA, TCI)) with 0.25 wt% photoinitiator TPO, which was replaced another 3 times for 24 h each. Then, the samples were removed from the solution and cured under a UV LED lamp for at least 30 min. For hydrogel-silica aerogel nanocomposites, after ethanol washing, the aged pure DLP printed wet gels were transferred to the precursor solution (80 wt% water from Milli-Q system, 19 wt% 2-hydroxyethyl acrylate (HEA, Acros), 1 wt% TEGDA) with 0.1 wt% photoinitiator lithium phenyl(2,4,6-trimethylbenzoyl)phosphinate (TCI), which was replaced another 3 times for 24 h each. Next, the samples were post-cured as above. In simulated drug loading-release experiments, 0.1 wt% disodium 4,4'-bis(2-sulfonatostyryl)biphenyl (TCI) as a model compound was added to the precursor solution and loaded into the nanocomposite during the subsequent light-curing process.

Characterization: The thermogravimetric curves were obtained from a thermogravimetric analyzer (TGA, Pyris 1, PerkinElmer) at 10 °C min^{-1} for silica aerogels and 5 °C min^{-1} for isocyanate-modified aerogels from 30-800 °C under air. The micromorphology was characterized by scanning electron microscopy (SEM, JSM-7500F, JEOL) and transmission electron microscopy (TEM, Talos F200X, FEI, accelerating voltage of 200 kV). The porous properties of aerogels were investigated by N_2 sorption/desorption isotherm measurements at 77 K obtained by a Tristar II 3020 surface area analyzer (Micromeritics Instrument Corporation). The specific surface area was determined using the BET method, and the pore size distribution was analyzed using the BJH method. The skeletal density was measured by He pycnometry using an automatic true density analyzer (Ultrapyc-5000, Anton Paar). The transmittance and absorbance were investigated using a UV–visible-NIR spectrometer (Lambda 950, Perkin Elmer) with a 150 mm integrating sphere. The thermal infrared imaging was performed by an infrared camera (TiS65, Fluke). Fourier transform infrared (FTIR) spectroscopy was performed on an FTIR spectrometer (Tensor 27, Bruker) with an

attenuated total reflection (ATR) attachment. For solid-state $^{13}C/^{29}Si$ nuclear magnetic resonance (NMR) spectroscopy, the powdered samples were packed in 4 mm ZrO_2 rotors, and the spectra were acquired by a Bruker Avance III 400 spectrometer (399.2 MHz for ^{1}H, 100.4 MHz for ^{13}C, and 79.3 MHz for ^{29}Si) using cross-polarization (CP), magic angle spinning (MAS) at a spin rate of 8 kHz, and a total suppression of sidebands (TOSS) sequence. The contact time for CP was 3 ms (^{13}C) and 5 ms (^{29}Si), with a recycle delay of 2 s, respectively. Adamantane was chosen as the external reference material (chemical shifts: 38.48 ppm). The thermal conductivity was determined by a Hot Disk 2500 S based on the transient plane source (TPS) method at room temperature. Disc-shaped samples larger than 24 mm in diameter and 3 mm in thickness were used. The alternating current (AC) impedance spectroscopy was measured by an electrochemical workstation (Autolab PGSTAT302N, Metrohm) at room temperature from 0.1 to 100 kHz with amplitude of 10 mV. At least three samples were measured to reduce the uncertainty of the results.

Rheological measurements were performed using a rheometer (MCR 302, Anton Paar) at room temperature. Real-time photorheology was measured in parallel plate mode (PP25, 25 mm in diameter), with a gap of 0.1 mm, shear frequency of 10 rad s^{-1}, and strain amplitude of 0.1%. The light source used was an Omnicure Series 1500 with a wavelength range of 320-500 nm and an output intensity of 26 mW cm^{-2}. To stabilize the system, the light was turned on 30 s after the start of the rheological test. The shear viscosity test was performed by a cylinder measuring system (CC27). In a single test, the shear rate increased logarithmically from 1 to 100 s^{-1}.

In the compression test, all samples were cylindrical with dimensions of approximately 6 mm in diameter and 6 mm in height, except for the ionogel and hydrogel matrix samples, which were approximately 7 mm in diameter and 11 mm in height. In the three-point bending (3PB) test, all samples were rectangular with dimensions of approximately 40 mm × 6.5 mm × 2.3 mm. The support span of DLP-printed silica aerogels in 3PB was 36 mm, and 34 mm for the rest samples. All tests were performed at a loading rate of 1 mm min^{-1} except at 5 mm min^{-1} for the 60-cycle compression test.

All tests were performed by a universal tensile machine (UTM-16555, Shenzhen Suns Technology) with a 1 kN force transducer (10 kN for the compression test of isocyanate-modified aerogels), except for the compression test of epoxy-silica aerogel nanocomposites, which was performed using another universal tensile machine (HS-100KN, Huahui Test Machine) with a 100 kN force transducer. At least three samples were tested at room temperature to reduce the uncertainty of the results.

Table S1. The formulations of the modified silica sols containing different ratios of precursors

Designation	V_{TEOS}/V_{MAPTMS}	W_{MAPTMS} (wt%)	W_{TEOS} (wt%)	$W_{MAPTMS+TEOS}$ (wt%)	n_{MAPTMS}/n_{TEOS} (mol%)
group 237	23/7 (69/21)	10.2	30.1	40.3	28.4
group 255	25/5 (75/15)	7.3	32.8	40.1	18.7
**group 273**	**27/3** (81/9)	**4.4**	**35.6**	**40**	**10.4**
**group 282**	**28/2** (84/6)	**2.9**	**36.9**	**39.8**	**6.6**
group 291	29/1 (87/3)	1.5	38.3	39.8	3.3

Table S2. The physical properties of the isocyanate-modified DLP-printed silica aerogels

Name	ρ_{bulk} (g cm^{-3})	$\rho_{skeletal}$ (g cm^{-3})	Porosity[a] (%)	V_{total}[b] (cm^3 g^{-1})	S_{BET} (m^2 g^{-1})	$V_{p,BJH}$ (cm^3 g^{-1})	$D_{p,BJH}$ (nm)
282-PU-1	0.535 ± 0.025	1.42	62.3	1.16	107	0.45	14.2
282-PU-2	0.714 ± 0.057	1.36	47.5	0.67	82	0.35	15.1
282-PU-3	0.871 ± 0.045	1.30	33.0	0.38	30	0.15	18.3

[a] Porosity was calculated by $1 - \rho_{bulk}/\rho_{skeletal}$

[b] V_{total} was calculated by $1/\rho_{bulk} - 1/\rho_{skeletal}$

As the content of isocyanate increased, the bulk density of the modified aerogel increased, the specific surface area (S_{BET}) and the pore volume (V_{total}) decreased, and the average pore size ($D_{p,BJH}$) increased. These results indicated that after isocyanate modification, the as-formed polymer coated the native skeleton of silica aerogel and filled the mesopores in the network. More detailed characterizations are shown in Figure S15.

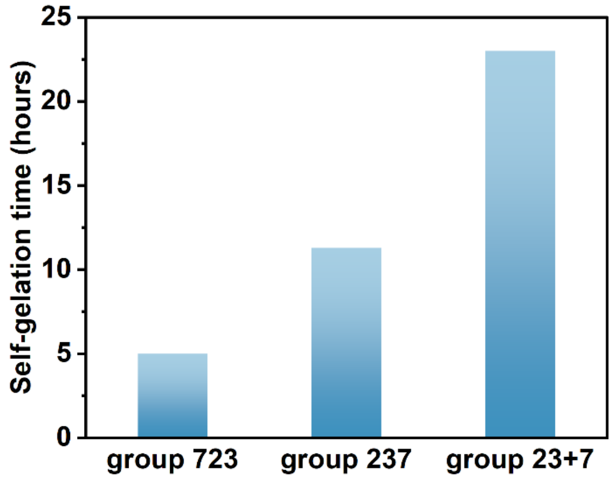

Figure S1. The self-gelation time (t_{sg}) of the silica sols prepared by different dosing orders of precursors. The corresponding reaction times for the preparation of the silica sols were as follows: 1) group 723, 24 h (MAPTMS) + 48 h (after addition of TEOS); 2) group 237, 24 h (TEOS) + 48 h (after addition of MAPTMS); and 3) group 23+7, 72 h (TEOS+MAPTMS). The self-gelation time was estimated by visual observation of the time required for the system to spontaneously undergo a sol-gel transition after the addition of HMTA.

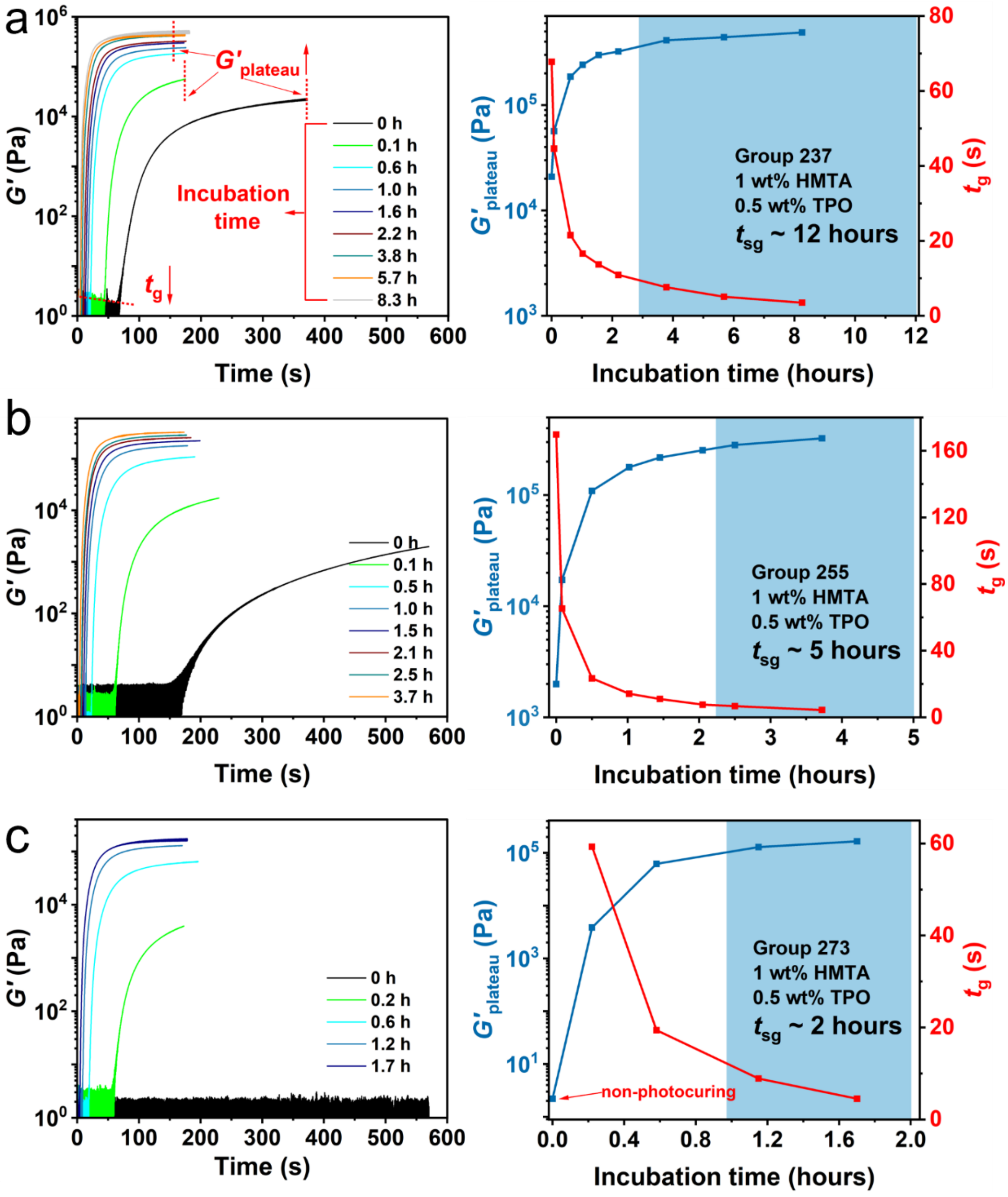

Figure S2. The photorheology properties of the acrylate-modified silica sols (from groups 237 to 273) at different incubation times after the addition of 1 wt% HMTA. The incubation time of 0 h indicates that the silica sol is under acidic conditions (without HMTA). Under acidic conditions, the photoinduced gelation time (t_g) increased when the concentration of MAPTMS (C_{MAPTMS}) was reduced. For group 273 in (c), no change in the storage modulus (G') was detected within 570 s after the light was turned on, indicating that the sols containing fewer acrylate groups were not photocrosslinked under acidic conditions. The blue areas in the figures show the stable

printing stages, with the features of relatively stable t_g and $G'_{plateau}$, that could be used for DLP.

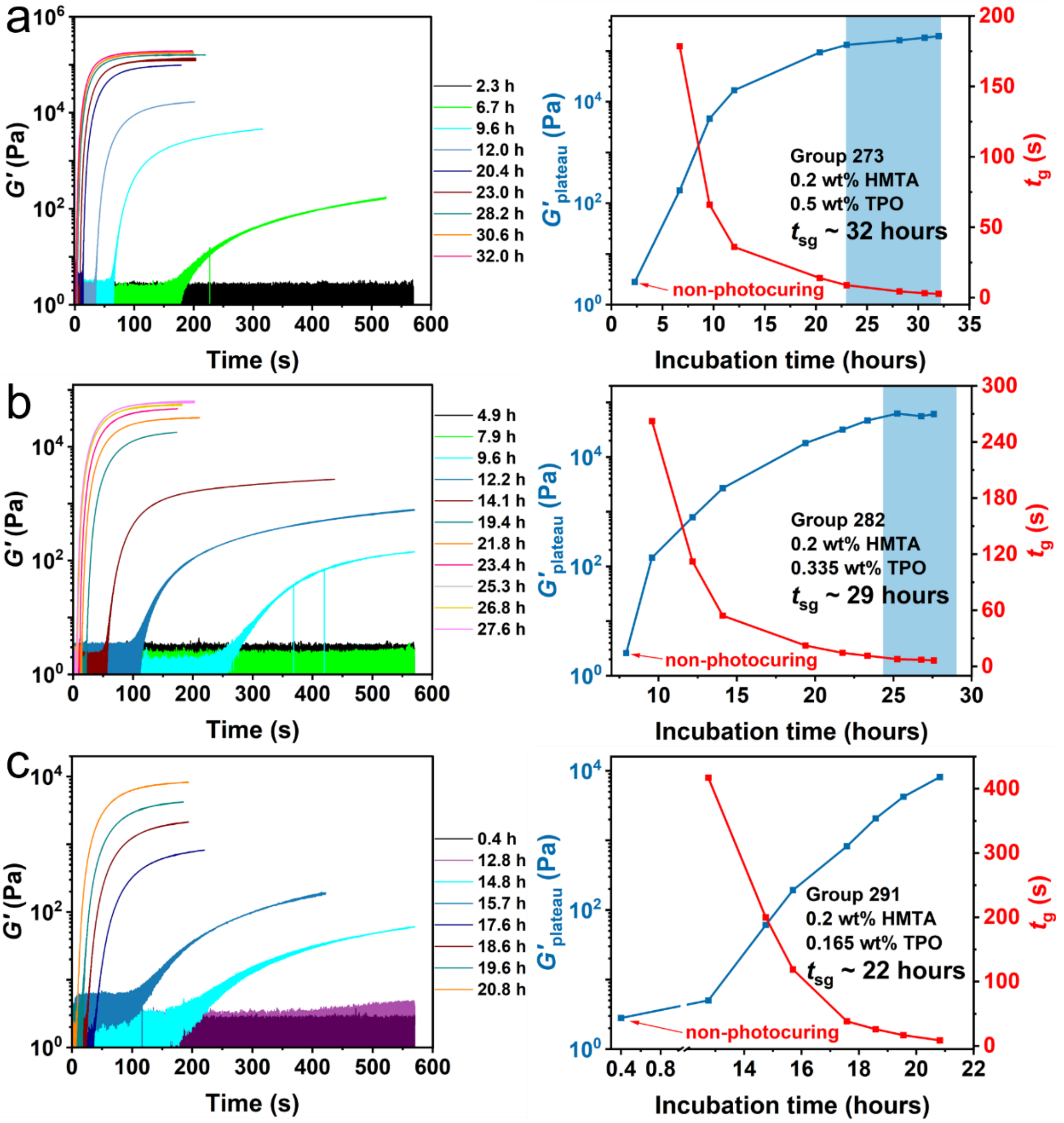

Figure S3. The photorheology properties of the acrylate-modified silica sols (from groups 273 to 291) at different incubation times after the addition of 0.2 wt% HMTA. Apparently, there was almost no stable printing stage (blue area) for group 291.

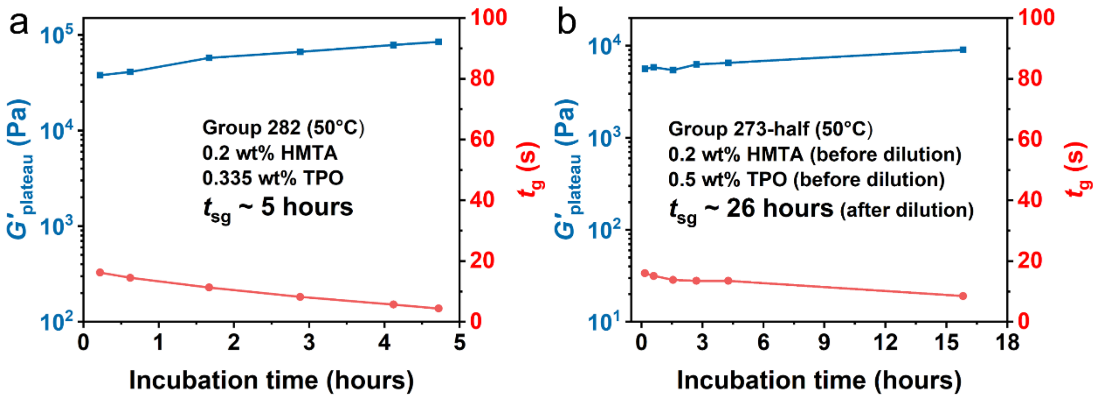

Figure S4. The evolution of the photorheology behaviors of group 282 and group 273-half during the stable printing stage for DLP. After the addition of HMTA (0.2 wt%), the sols of 282 and group 273-half were heated at 50 °C for 2 and 3 h, respectively, to accelerate the condensation reaction. The self-assembly of the modified silica sol was promoted, and the incubation time decreased as a result.

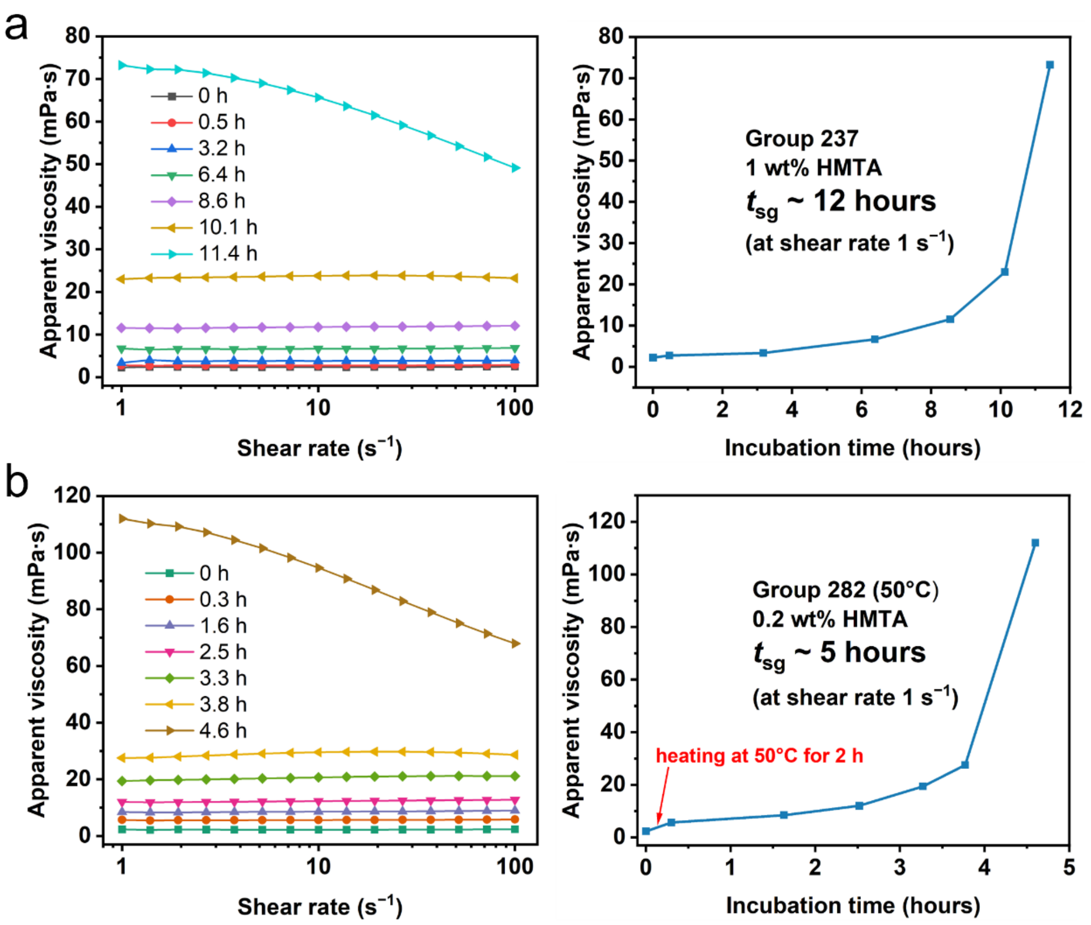

Figure S5. (a) The trend of the apparent viscosity of group 237 with incubation time (1 wt% HMTA). (b) The trend of the apparent viscosity of group 282 at the stable printing stage with incubation time (after the addition of 0.2 wt% HMTA and heating at 50 °C for 2 h). When approaching the spontaneous gelation point, the aggregates grew larger through Si-O-Si bridge bonding and entangled, thus exhibiting shear-thinning behavior at high shear rates.

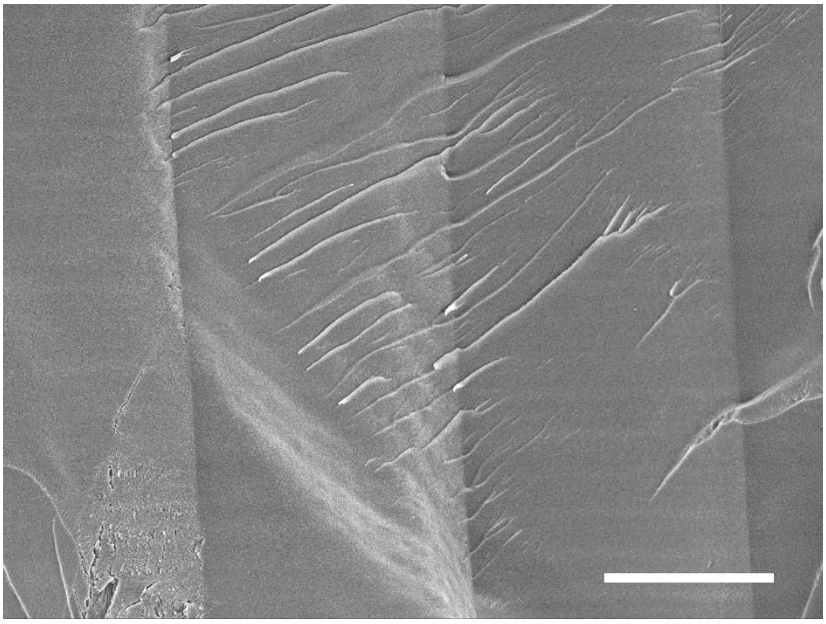

Figure S6. Scanning electron microscopy (SEM) image of the layers in the printed object from group 273-half. Scale bar: 100 μm. The preset layer thickness was 200 μm, and the actual layer thickness after drying was approximately 160 μm, indicating ~20% linear shrinkage, which was attributed to the free volume reduction due to polymerization in 3D printing and subsequent aging.

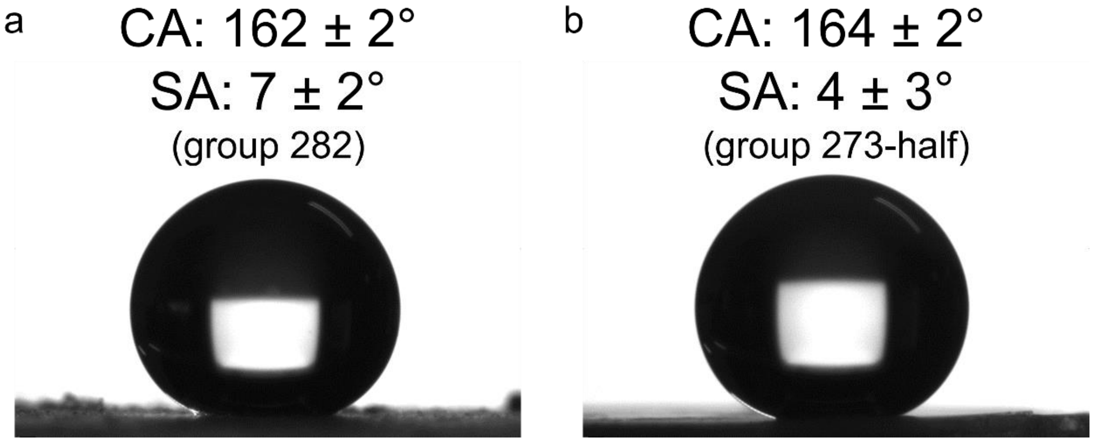

Figure S7. Images of water droplets on the surface of DLP-printed objects from group 282 (a) and 273-half (b).

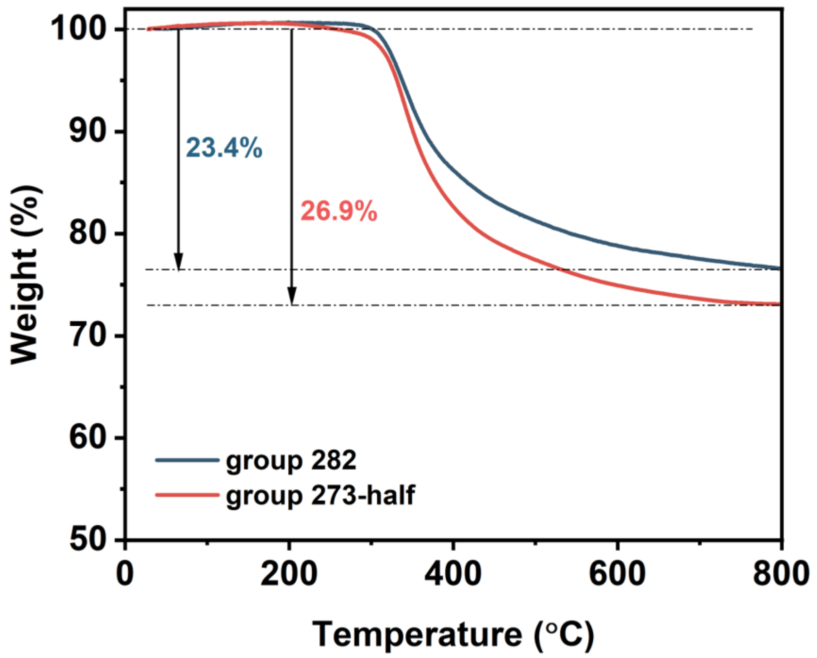

Figure S8. TGA data for DLP-printed silica aerogels after hydrophobization. The weight loss at > 300 °C was mainly derived from the organic groups of MAPTMS and ≡Si-CH_3.[3, 4]

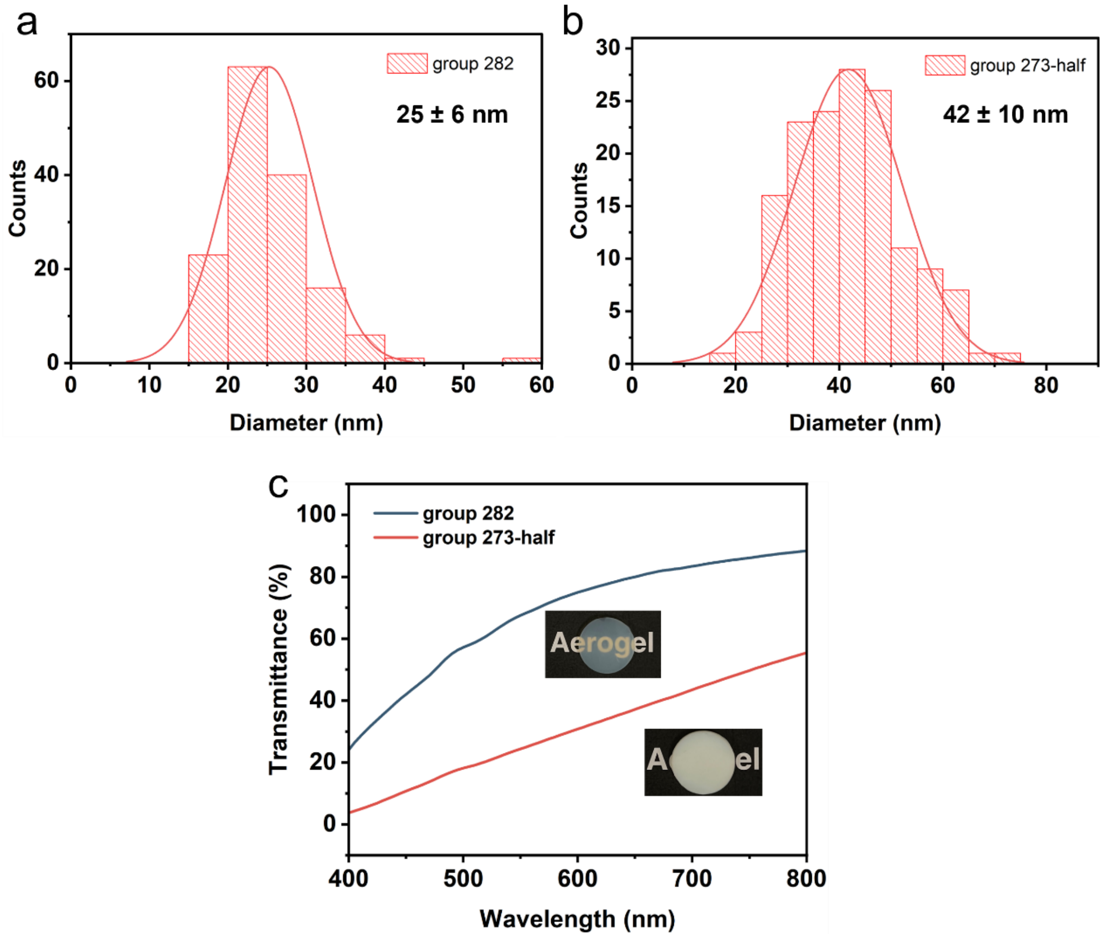

Figure S9. Size distributions of the skeletons of DLP-printed aerogels from groups 282 (a) and 273-half (b). The size was evaluated by Image J from SEM images. (c) The transmittance of DLP-printed discs from group 282 (4.0 mm thick) and group 273-half (4.6 mm thick) and the corresponding digital photographs. The transmittance of sample 273-half decreased since $\alpha \propto d^3$ where α is the extinction coefficient and d is the scatterer size, i.e., the skeleton size, according to Rayleigh-Gans theory.[5]

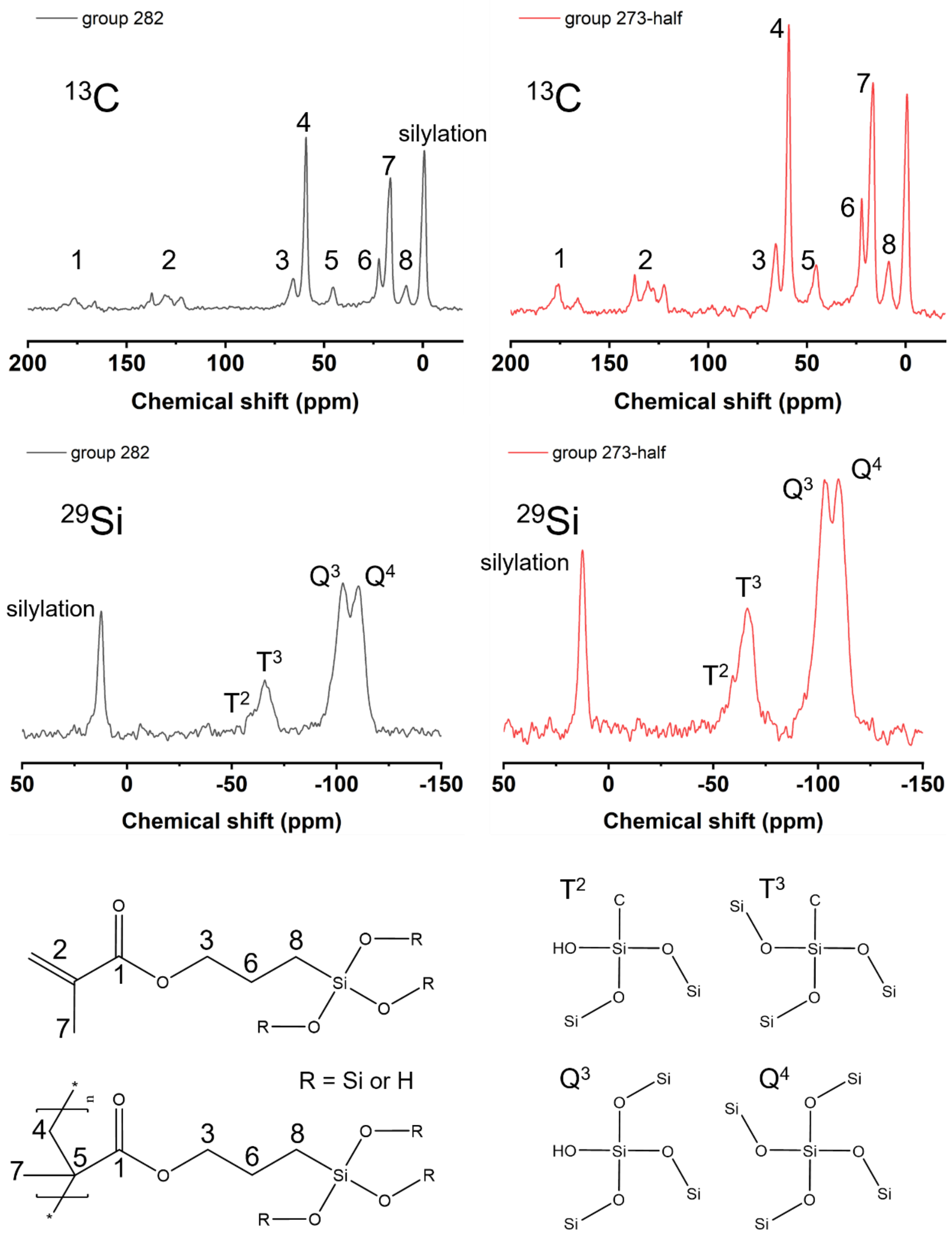

Figure S10. Solid-state ^{13}C CP/TOSS and ^{29}Si CP/MAS NMR spectra of the DLP-printed silica aerogels and the corresponding peak attribution.[6] The Si-O-Si bond angles (θ) were 148.3° and 146.9° in groups 282 and 273-half aerogels, respectively, calculated from $\delta_{Q^4}(\theta) = -93.12 + 8.66\,cos(\theta) - 22.27\,cos(2\theta)$ when $\delta_{Q}{}^{4} < 120$

ppm where $\delta_{Q}{}^{4}$ is the chemical shift of the characteristic peak of the fully condensed Si atom of Q^4 in the ^{29}Si solid-state NMR spectrum.[3, 7]

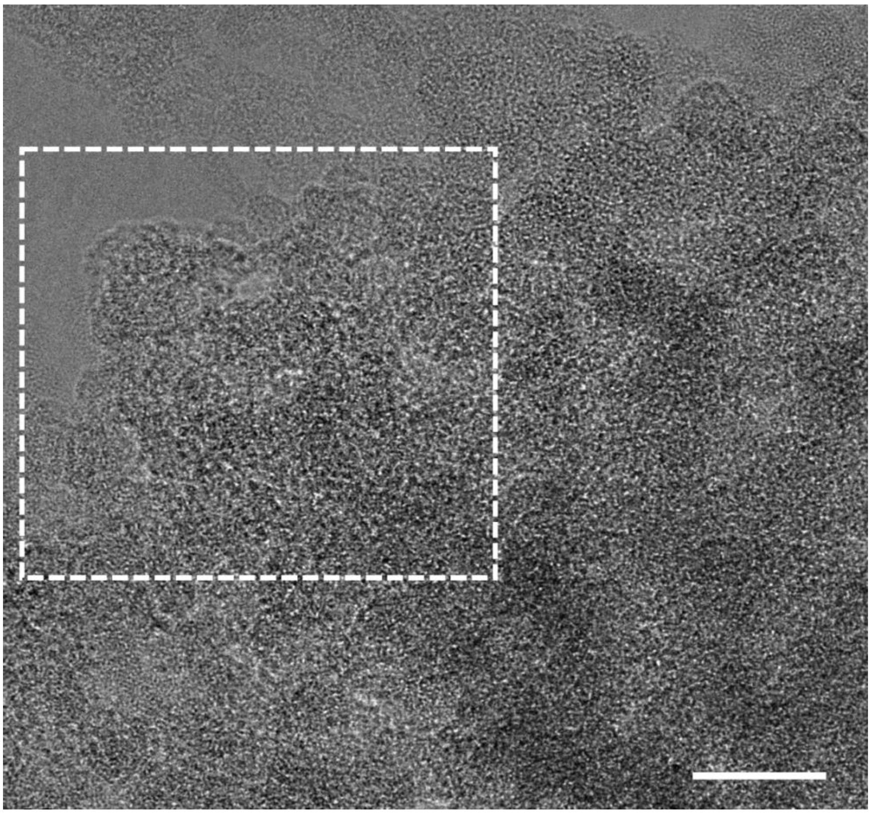

Figure S11. High-resolution transmission electron microscopy (HRTEM) image of the DLP-printed silica aerogel from group 273-half (scale bar: 10 nm). No significant phase interface (difference in imaging contrast) between the organic polymer region (carbon atom enrichment) and the silica region (silicon atom enrichment) could be observed on the scale of approximately 10 nm, indicating that a small amount of polymethacrylate was uniformly distributed in the silica network of the aerogels.

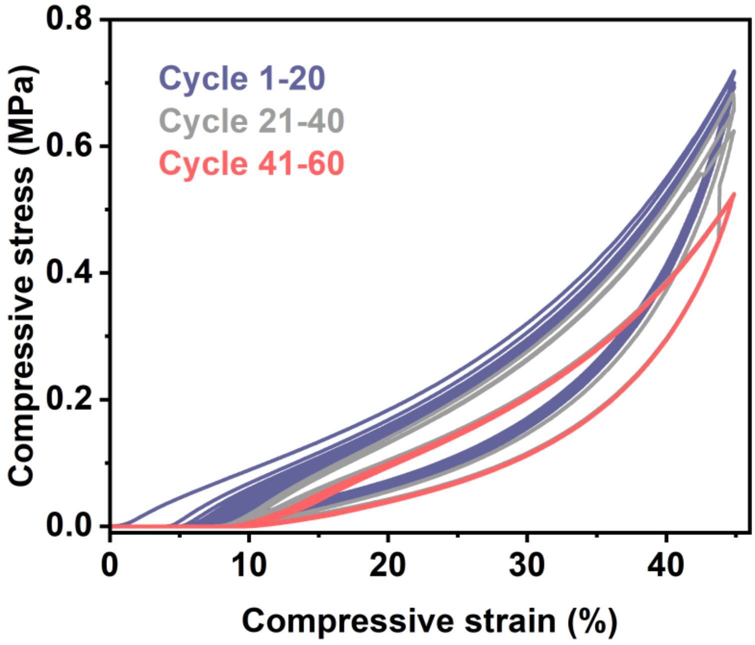

Figure S12. Uniaxial compression-decompression testing of 60 cycles for the DLP-printed silica aerogel from group 273-half.

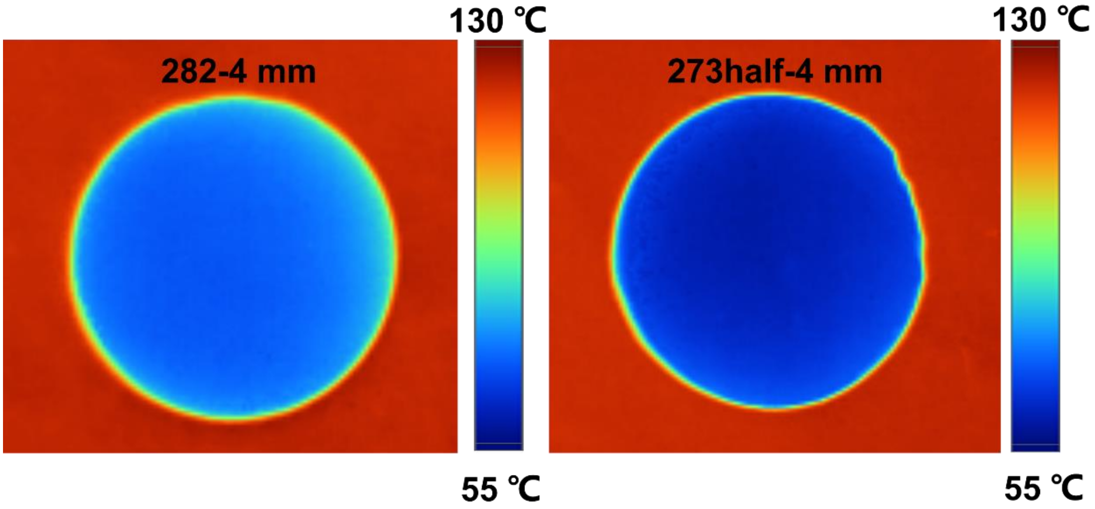

Figure S13. Infrared images of 4 mm thick 3D-printed disk-shaped samples placed on a heated copper plate at approximately 120 °C for 0.5 h to reach thermal equilibrium. The surface of the copper plate was sprayed with a black graphite coating (Graphit 33, Kontakt Chemie) to ensure sufficient infrared emissivity.

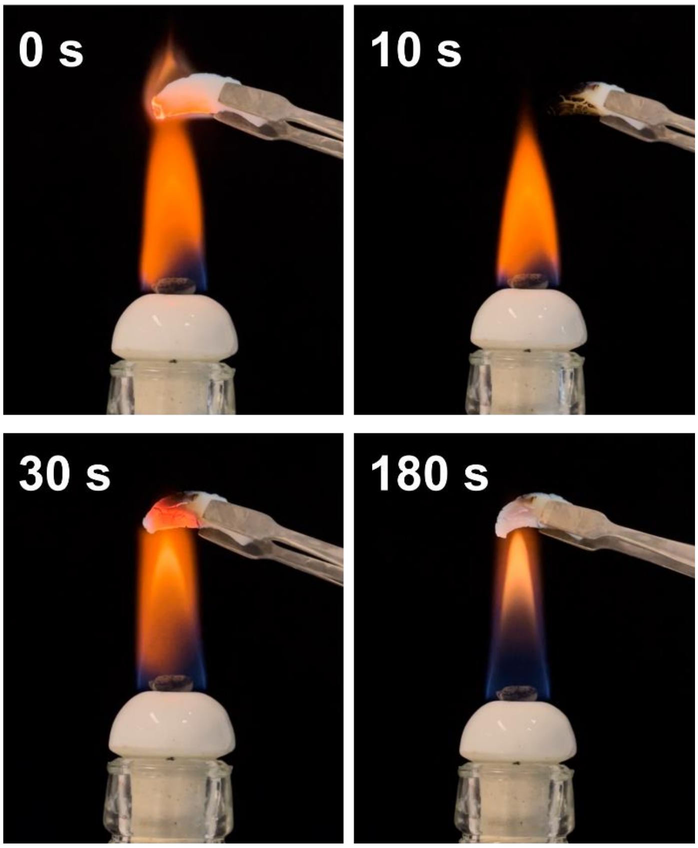

Figure S14. Photographs of an alcohol lamp flame during an ablation test of a printed aerogel sample from group 273-half.

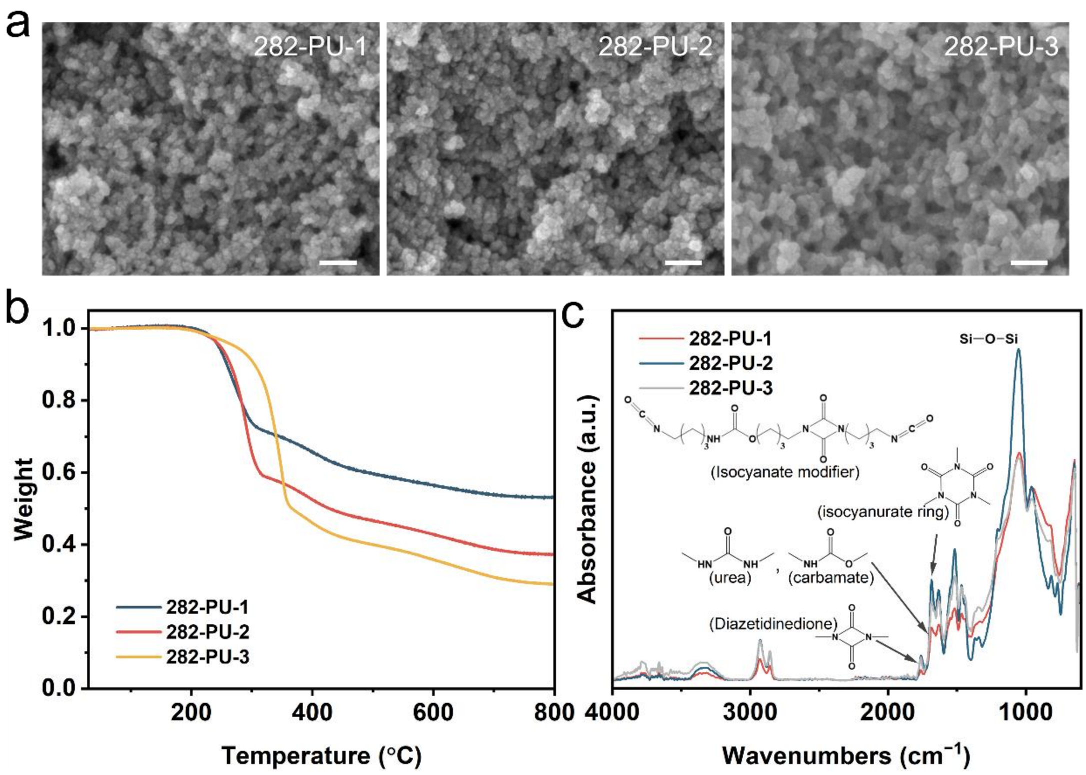

Figure S15. (a) SEM photographs of the isocyanate-modified DLP-printed silica aerogels from group 282 with different bulk densities, i.e., different isocyanate contents, scale bar: 100 nm; (b) TGA curves of the modified aerogels; (c) Attenuated total reflectance-Fourier transform infrared spectroscopy (ATR-FTIR) spectra of the modified aerogels. SEM images showed that the pearl-necklace-like structure was preserved, but the skeleton was significantly thicker with increasing isocyanate content, which was consistent with the results of the N_2 sorption/desorption test, as shown in Table S2. The ATR-FTIR spectra confirmed the presence of diazetidinedione (~1767 cm^{-1}, from isocyanate) and carbamate (or urea group/isocyanurate ring with overlapping absorption peak positions, ~1690 cm^{-1}) in all modified aerogels.[2, 8] The carbamate was formed by the reaction of silanol group with isocyanate. The urea group was formed by the reaction between isocyanates after being hydrolyzed into an amine group by water confined on the skeleton surface. The isocyanurate ring was formed by the condensation of the isocyanate). Besides, Si-O-Si bonds (~1078 cm^{-1}) were present in all modified aerogels, and unreacted isocyanates (~2270 cm^{-1}) were not observed.

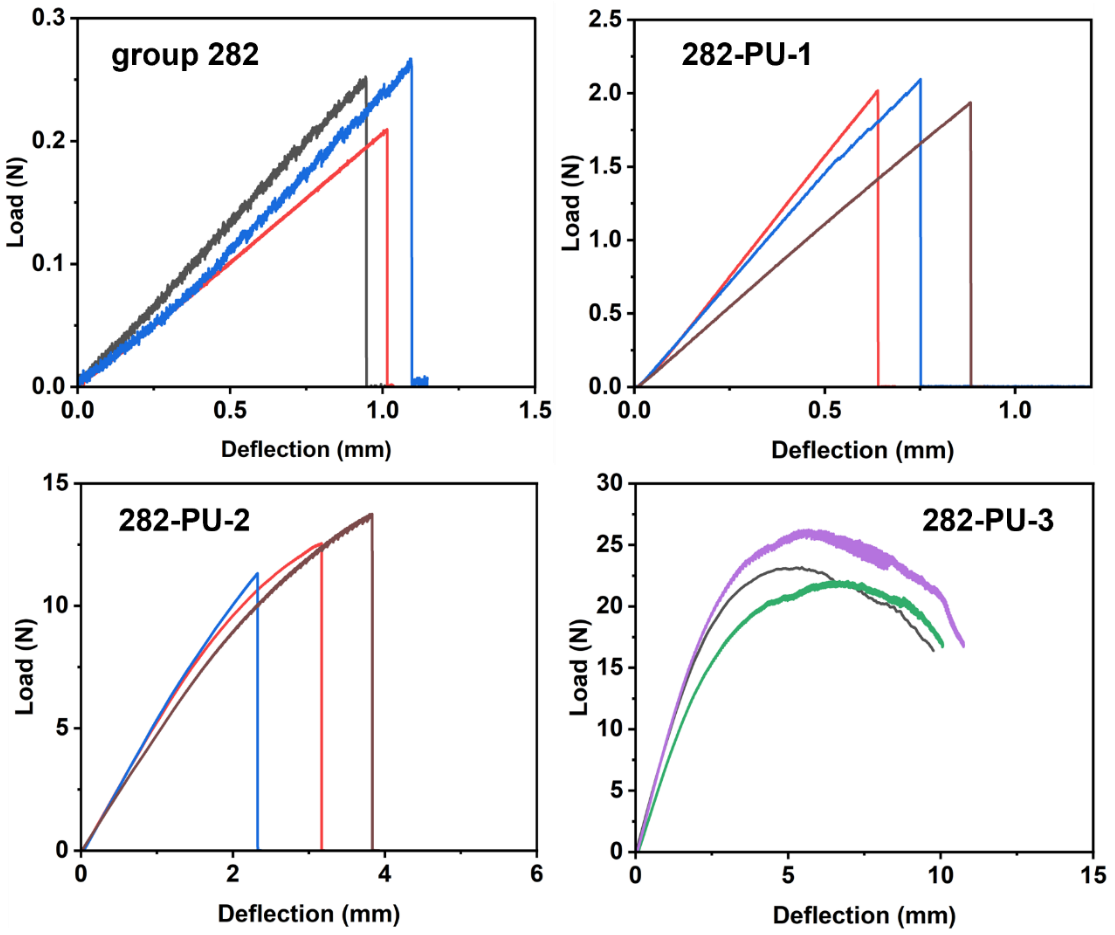

Figure S16. Typical load-deflection curves of the three-point bending test of the pristine and isocyanate-modified DLP-printed silica aerogels with different bulk densities.

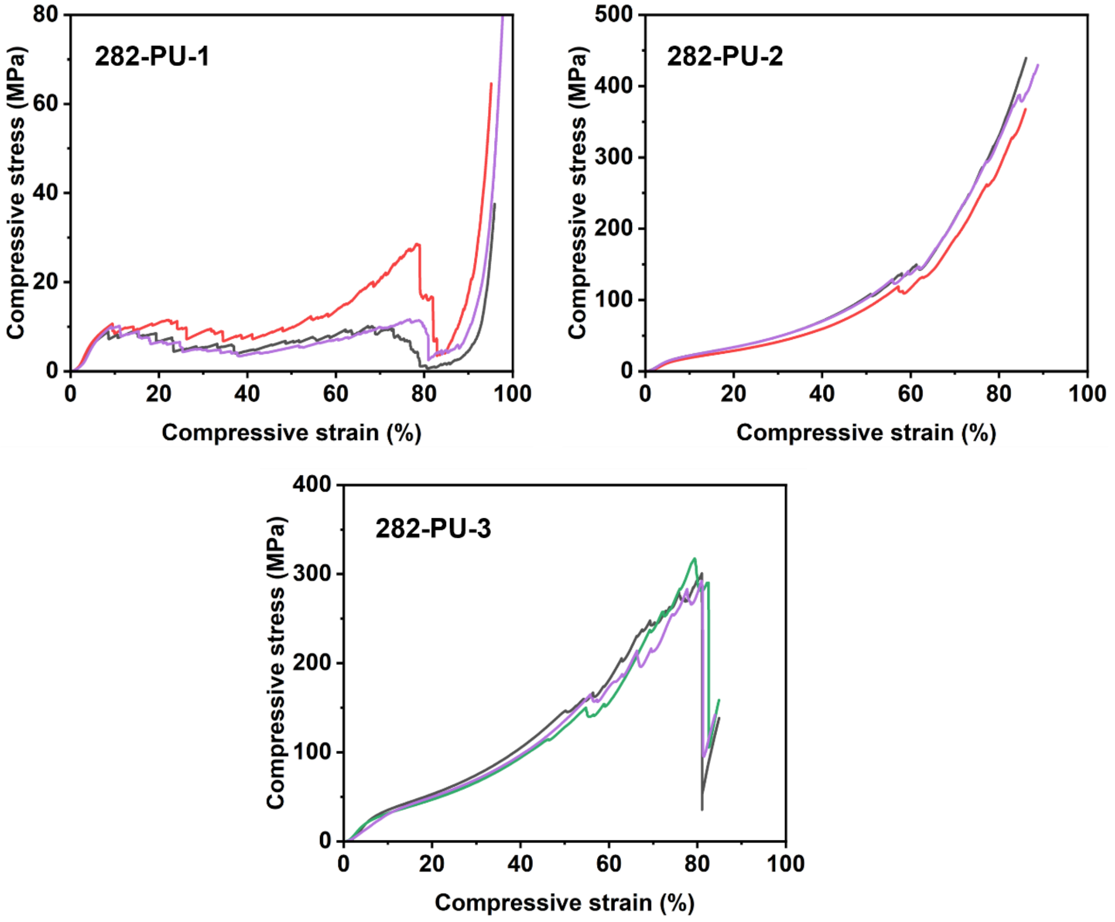

Figure S17. Typical stress–strain curves of the uniaxial compression test of 282-PU aerogels with different bulk densities.

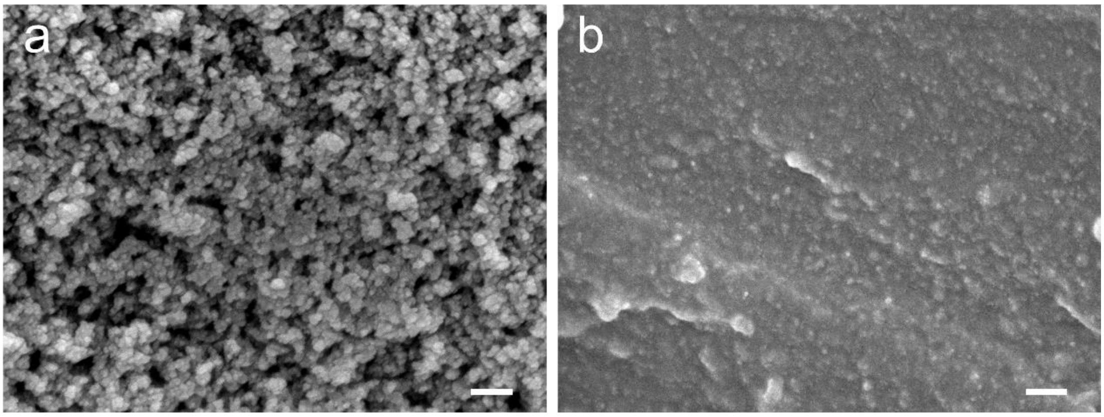

Figure S18. SEM photographs of the internal morphology of 282-PU-1 (a) and 282-PU-EP-1 (b). After infiltrating epoxy into 282-PU-1 and curing, no pores were observed inside the resultant composite. Scale bar: 100 nm.

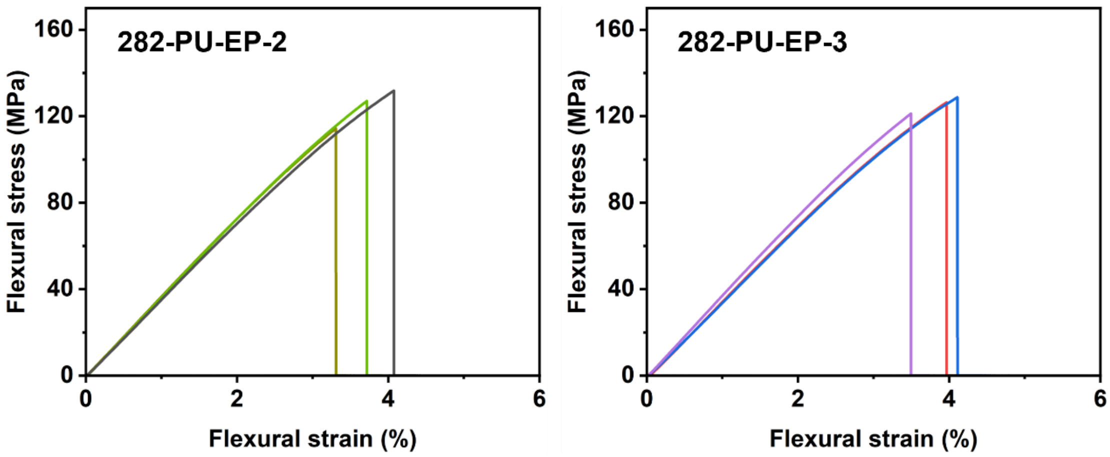

Figure S19. Typical stress–strain curves of the three-point bending test of 282-PU-EP-2 and 282-PU-EP-3 nanocomposites.

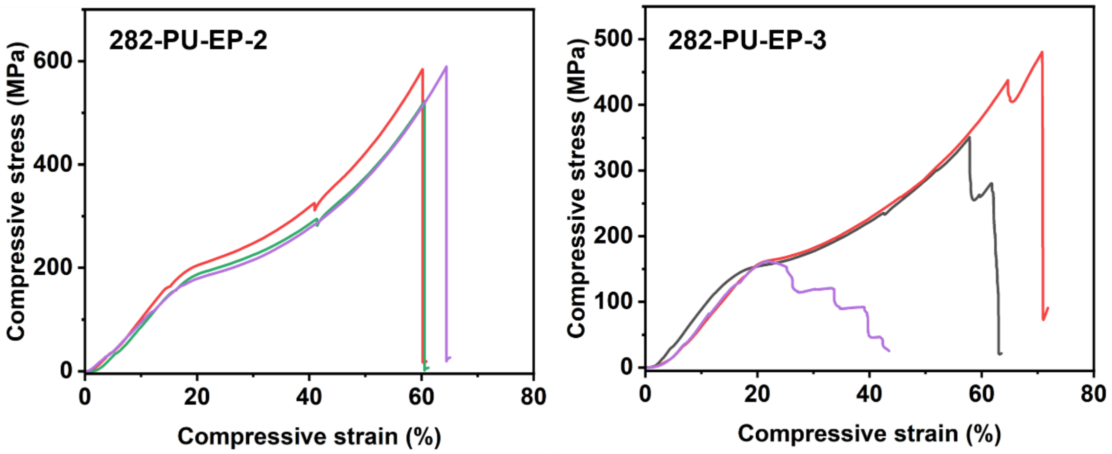

Figure S20. Typical stress–strain curves of the uniaxial compression test of 282-PU-EP-2 and 282-PU-EP-3 nanocomposites.

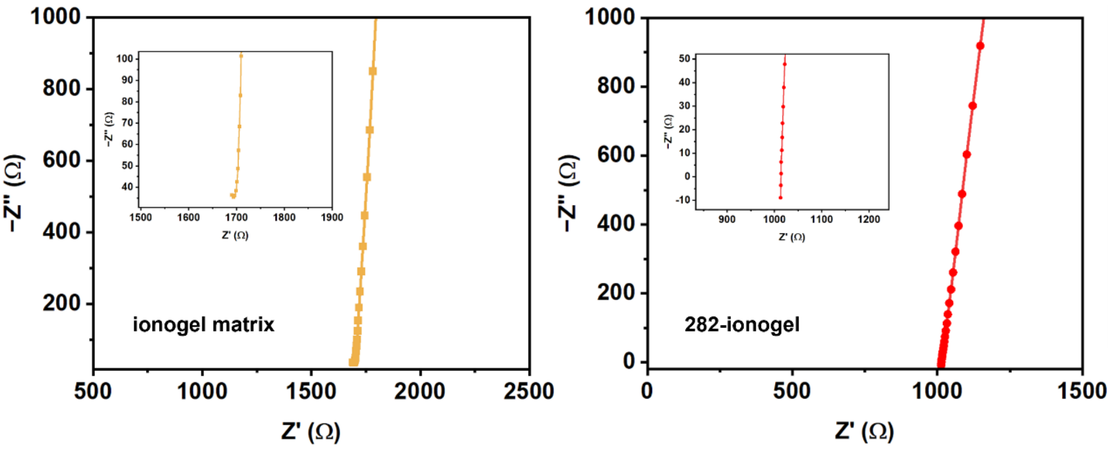

Figure S21. Nyquist plots demonstrating the impedance of the ionogel matrix and 282-ionogel nanocomposite. The ionic conductivity was obtained from the above impedance spectra. All samples used for this test were the same size as those used in the uniaxial compression test.

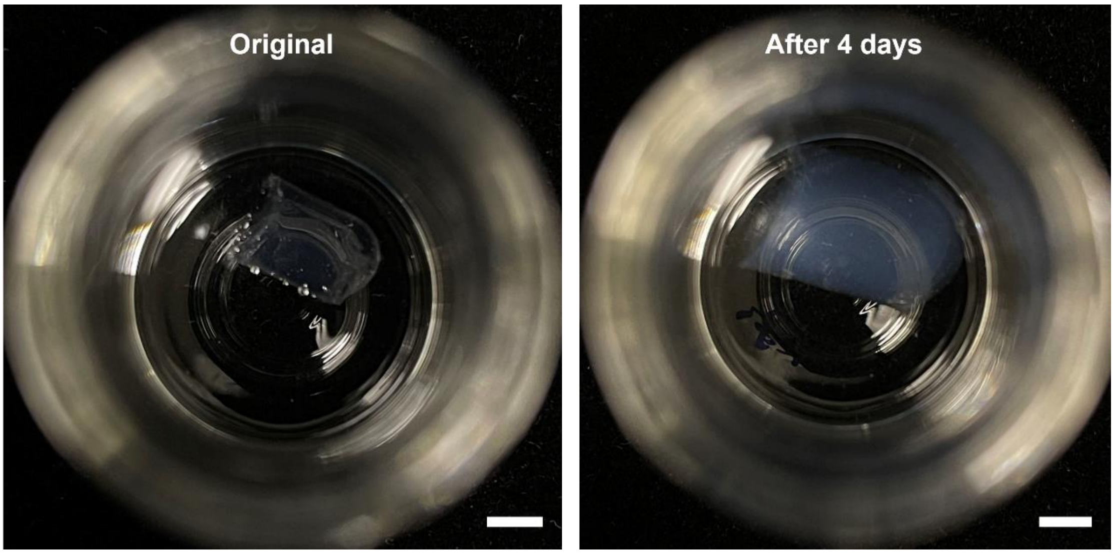

Figure S22. The swelling behavior of the hydrogel matrix in pure water. Scale bar: 5 mm.

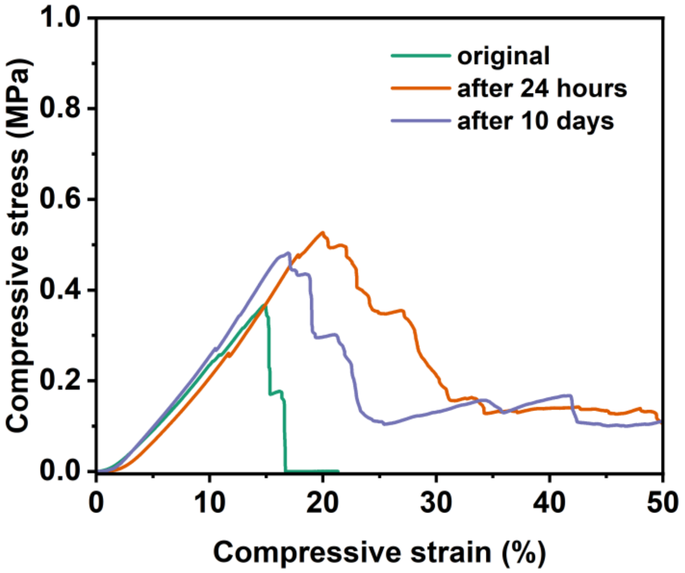

Figure S23. Compression properties of the 273-half-hydrogel composites after soaking in pure water for different periods.

References

1. E. S. Farrell, N. Ganonyan, I. Cooperstein, M. Y. Moshkovitz, Y. Amouyal, D. Avnir, S. Magdassi, *Appl. Mater. Today* **2021**, *24*, 101083.
2. G. Zhang, A. Dass, A.-M. M. Rawashdeh, J. Thomas, J. A. Counsil, C. Sotiriou-Leventis, E. F. Fabrizio, F. Ilhan, P. Vassilaras, D. A. Scheiman, L. McCorkle, A. Palczer, J. C. Johnston, M. A. Meador, N. Leventis, *J. Non-Cryst. Solids* **2004**, *350*, 152.
3. A. L. B. Maçon, S. J. Page, J. J. Chung, N. Amdursky, M. M. Stevens, J. V. M. Weaver, J. V. Hanna, J. R. Jones, *Phys. Chem. Chem. Phys.* **2015**, *17*, 29124.
4. Z. Li, X. Cheng, S. He, X. Shi, L. Gong, H. Zhang, *Compos. Pt. A-Appl. Sci. Manuf.* **2016**, *84*, 316.
5. X. Ji, Y. Du, X. Zhang, *Adv. Mater.* **2022**, *34*, 2107168.
6. A. P. A. M. Eijkelenboom, W. E. J. R. Maas, W. S. Veeman, G. H. W. Buning, J. M. J. Vankan, *Macromolecules* **1992**, *25*, 4511.
7. F. Mauri, A. Pasquarello, B. G. Pfrommer, Y.-G. Yoon, S. G. Louie, *Phys. Rev. B* **2000**, *62*, R4786.
8. A. Katti, N. Shimpi, S. Roy, H. B. Lu, E. F. Fabrizio, A. Dass, L. A. Capadona, N. Leventis, *Chem. Mater.* **2006**, *18*, 285.
